# Trends in antimicrobial susceptibility for azithromycin and ceftriaxone in *Neisseria gonorrhoeae* isolates in Amsterdam, the Netherlands, between 2012 and 2015

**DOI:** 10.2807/1560-7917.ES.2017.22.1.30431

**Published:** 2017-01-05

**Authors:** Carolien M Wind, Maarten F Schim van der Loeff, Alje P van Dam, Henry JC de Vries, Jannie J van der Helm

**Affiliations:** 1 STI Outpatient Clinic, Department of Infectious Diseases Public Health Service Amsterdam, Amsterdam, the Netherlands; 2 Department of Dermatology, Academic Medical Center, University of Amsterdam, Amsterdam, the Netherlands; 3 Department of Infectious Diseases, Public Health Service Amsterdam, Amsterdam, the Netherlands; 4 Center for Infection and Immunity Amsterdam, Academic Medical Center, University of Amsterdam, Amsterdam, the Netherlands; 5 Public Health Laboratory, Public Health Service Amsterdam, Amsterdam, the Netherlands; 6 Department of Medical Microbiology, Onze Lieve Vrouwe Gasthuis General Hospital, Amsterdam, the Netherlands

**Keywords:** *Neisseria gonorrhoeae*, antimicrobial resistance, susceptibility, azithromycin, ceftriaxone

## Abstract

Resistance of *Neisseria gonorrhoeae* to azithromycin and ceftriaxone has been increasing in the past years. This is of concern since the combination of these antimicrobials is recommended as the first-line treatment option in most guidelines. To analyse trends in antimicrobial resistance, we retrospectively selected all consultations with a positive *N. gonorrhoeae* culture at the sexually transmitted infection clinic, Amsterdam, the Netherlands, from January 2012 through September 2015. Minimum inhibitory concentrations (MICs) for azithromycin and ceftriaxone were analysed per year, and determinants associated with decreased susceptibility to azithromycin (MIC > 0.25 mg/L) or ceftriaxone (MIC > 0.032 mg/L) were assessed. Between 2012 and 2015 azithromycin resistance (MIC > 0.5 mg/L) was around 1.2%, the percentage of isolates with intermediate MICs (> 0.25 and ≤ 0.5 mg/L) increased from 3.7% in 2012, to 8.6% in 2015. Determinants associated with decreased azithromycin susceptibility were, for men who have sex with men (MSM), infections diagnosed in the year 2014, two infected sites, and HIV status (HIV; associated with less decreased susceptibility); for heterosexuals this was having ≥ 10 sex partners (in previous six months). Although no ceftriaxone resistance (MIC > 0.125 mg/L) was observed during the study period, the proportion of isolates with decreased ceftriaxone susceptibility increased from 3.6% in 2012, to 8.4% in 2015. Determinants associated with decreased ceftriaxone susceptibility were, for MSM, infections diagnosed in 2014, and pharyngeal infections; and for heterosexuals, infections diagnosed in 2014 or 2015, being of female sex, and having ≥ 10 sex partners. Continued decrease of azithromycin and ceftriaxone susceptibility will threaten future treatment of gonorrhoea. Therefore, new treatment strategies are warranted.

## Introduction

Since penicillin became available in the 1940s, *Neisseria gonorrhoeae* infection has become a treatable sexually transmitted infection (STI) [1]. Yet successful eradication is hampered by emerging resistance to all first-line antibiotics used so far. Latest in this trend are resistance and treatment failures to extended-spectrum cephalosporins (ESC) [1,2]. We reported an increase in ESC-resistant *N. gonorrhoeae* among men who have sex with men (MSM) in Amsterdam, the Netherlands, between 2006 and 2008 [3]. To halt the development and spread of resistance, international gonorrhoea guidelines recommend dual therapy consisting of ceftriaxone (an ESC) and azithromycin [4-6]. Dual therapy is also effective against *Chlamydia trachomatis*, which frequently coincides with gonorrhoea [4]. However, resistance and treatment failures have been documented for both drugs [7-13]. Taking the historical course of emerging antimicrobial-resistant gonorrhoea strains into account, without additional measures, a further decrease in ceftriaxone and azithromycin susceptibility is to be expected [1]. Moreover, high level azithromycin-resistant gonorrhoea has been reported in the United Kingdom (UK) since 2015 [9]. In addition, the first treatment failure on dual therapy of azithromycin and ceftriaxone was reported in 2016 [14]. The World Health Organization (WHO) recommends abandoning an antibiotic as first-line treatment once the prevalence of resistant strains in the population exceeds 5% [15]. Surveillance is essential to monitor this development. Therefore, we analysed the susceptibility to azithromycin and ceftriaxone of *N. gonorrhoeae* isolates among attendees of the STI Outpatient Clinic in Amsterdam, the Netherlands, between 2012 and 2015. We also assessed which determinants were associated with decreased susceptibility.

## Methods

### Study population

The STI Outpatient Clinic in Amsterdam, is the largest centre for STI care in the Netherlands, with up to 40,000 consultations each year [16]. We test and treat (free of charge) patients who: are younger than 25 years-old, commercial sex workers, clients of commercial sex workers, MSM, have ≥ 3 sex partners in the previous six months, were notified of an STI by a sex partner, have STI-related complaints, are of non-West-European origin, or are of non-North-American origin.

Dual therapy for gonorrhoea is not recommended in the Netherlands, instead ceftriaxone 500 mg is used, and azithromycin is added only in case of a suspected or proven coinfection with *C. trachomatis* [17]. This single treatment alternative is supported by the 2016 WHO gonorrhoea treatment guideline [6].

For this study, we included consultations from January 2012 through September 2015, with a positive *N. gonorrhoeae* culture, and available minimum inhibitory concentrations (MICs) for azithromycin and ceftriaxone. Per consultation, defined as all visits that are part of a new request for healthcare, a patient could be infected at up to four anatomical sites (cervix/vagina, pharynx, rectum, and urethra). Samples were collected from any site upon risk assessment; rectal and pharyngeal samples were not obtained from heterosexual males. When more than one culture was obtained during a single consultation, we included the one with the highest MIC for either azithromycin or ceftriaxone. In case of equal MICs at different anatomical sites, we gave priority in the following order: pharynx, cervix/vagina, rectum, and urethra. All analyses were performed using isolates collected during individual consultations, therefore some patients were included more than once. Patient and clinical characteristics were obtained from the electronic patient file. Syphilis status (past and active) was based on *Treponema pallidum* particle agglutination (TPPA) and rapid plasma reagin (RPR) testing, human immunodeficiency virus (HIV)-positivity was based on HIV-antibodies, and coinfection with *C. trachomatis* was diagnosed using a nucleic acid amplification test (NAAT) [3]. As this was a retrospective cohort study using only routinely obtained data, no ethical clearance or informed consent was required.

### Antimicrobial susceptibility testing

Up to May 2014, direct *N. gonorrhoeae* cultures instead of NAATs, were routinely obtained from urogenital and rectal sites, if patients met at least one of the following criteria: being MSM, having STI-related symptoms, being notified of gonorrhoea by a sex partner, or performing sex work. In addition, cultures were obtained from patients, who did not have any of the prior-described criteria for culture but had a positive NAAT for *N. gonorrhoeae*. Pharyngeal sites were primarily tested using NAAT, and cultures were obtained in case of positive results. From May 2014 onward this policy was changed, and NAAT was used as the routine test for gonorrhoea diagnosis in all patients and all anatomical sites. Cultures were obtained if a patient had symptoms suggestive of gonorrhoea, and intracellular Gram-negative diplococci had been identified in a Gram-stained smear, or if the NAAT was positive for gonorrhoea. In case of a positive culture for *N. gonorrhoeae*, antimicrobial susceptibility testing was routinely performed at the Public Health Laboratory in Amsterdam, the Netherlands [18]. MICs for azithromycin, cefixime, cefotaxime, ceftriaxone and ciprofloxacin were determined using Etests according to the manufacturer’s instructions (bioMérieux SA, Marcy-l’Étoile, France). For this study MIC data were obtained as recorded in the electronic laboratory patient files. To determine resistance we used the European committee on antimicrobial susceptibility testing (EUCAST) breakpoints [19]. For azithromycin we categorised MIC values into susceptible (MIC ≤ 0.25 mg/L), intermediate (MIC > 0.25 and ≤ 0.5 mg/L), and resistant (MIC > 0.5 mg/L). For ceftriaxone, cefixime and cefotaxime we categorised MICs into susceptible (MIC ≤ 0.125 mg/L) and resistant (MIC > 0.125 mg/L). For ciprofloxacin we categorised MICs into susceptible (MIC ≤ 0.06 mg/L) and resistant (MIC > 0.06 mg/L).

### Statistical analyses

Baseline characteristics were compared for MSM and heterosexuals using *X*^2^, Fisher exact, or Kruskal–Wallis tests. The prevalence of antimicrobial resistance in our population is still very low, and we could not determine associations with resistance. Therefore, we used not resistance, but decreased susceptibility as endpoint in the analyses. Decreased susceptibility was determined for azithromycin as MIC > 0.25 mg/L, and for ceftriaxone as MIC > 0.032 mg/L (the epidemiological cut-off as reported by EUCAST) [19]. Mean MICs were calculated as geometric means. To assess determinants associated with decreased susceptibility we performed logistic regression analyses. Since sexual orientation is highly correlated with many other variables, such as anatomical site, origin, age, and coinfections like HIV, syphilis and *C. trachomatis*, we performed separate analyses for MSM and heterosexuals. All determinants that were associated in the univariable analysis (p < 0.1) were included in the multivariable analysis, using backward selection. As our main category of interest for trend analysis, year of infection was always included in the model. Also sex (for heterosexuals only) and age were always included in the model. In the multivariable analysis statistical significance was determined as p < 0.05. All analyses were performed using Stata (version 13; StataCorp, College Station, Texas).

## Results

Gonorrhoea was diagnosed at our STI Clinic in 5,431 consultations from January 2012 through September 2015. We excluded 2,280 consultations in which a gonorrhoea diagnosis was based on results of a NAAT or a Gram-stained smear, but a *N. gonorrhoeae* culture was not performed (n = 653), was not positive (n = 1,590), or because no susceptibility data were available (n = 37). This resulted in 3,151 included consultations, from 2,573 individual patients. The majority of patients (n = 2,573) were included only once; 408 patients (13.0%) were included twice, 109 patients (3.5%) were included three times, and 61 patients (1.9%) were included with four to eight episodes. Of the 578 patients who were included more than once, 522 (90.3%) were MSM.

### Baseline characteristics of patients

Of the 3,151 included isolates, 2,318 (73.6%) were from MSM, and 833 (26.4%) were from heterosexual patients, of which 436 (52.3%) were from males and 397 (47.7%) were from females (Table 1). The median age was 34 years (interquartile range (IQR): 26–43) for MSM, and 23 years (IQR: 20–28) for heterosexuals. The majority of MSM were of Dutch origin (n = 1,347 isolates, 58.1%), while among heterosexuals the largest group was of Surinamese origin (n = 342 isolates, 41.1%), followed by 158 isolates of Dutch origin (19.0%). Heterosexuals were more likely to be symptomatic (n = 559, 67.1%) compared with MSM (n = 1,249, 53.9%, p < 0.001). The median number of sex partners in the previous six months was eight for MSM (IQR: 4–15), and three for heterosexuals (IQR: 2–5). While MSM were more likely to be HIV-positive (n = 900, 38.8%), or have (ever had) syphilis (n = 752, 32.4%) compared with heterosexuals (n = 7, 0.8%, and n = 14, 1.7%; p < 0.001 for both), they were less likely to be coinfected with *C. trachomatis* (n = 502, 21.7% for MSM, and n = 373, 44.8%, for heterosexuals, p < 0.001). Among the 2,318 MSM, the majority of isolates were from the rectum (56.2%), while 11.6% were from the pharynx, and 90.5% (n = 2,098) had only one culture positive site. Among heterosexuals the majority of isolates were from the urethra (54.1%) or the cervix/vagina (31.0%).

**Table 1:** Baseline characteristics of included consultations with culture positive *Neisseria gonorrhoeae*, at the STI Outpatient Clinic Amsterdam, the Netherlands, January 2012–September 2015 (n = 3,151 consultations)

Characteristic	MSM n (%)^a^	Heterosexual n (%)^a^	P
Isolates	2,318 (73.6)	833 (26.4)	NA
Year of diagnosis			
2012	633 (27.3)	286 (34.3)	0.001
2013	621 (26.8)	200 (24.0)	
2014	614 (26.5)	214 (25.7)	
2015^b^	450 (19.4)	133 (16.0)	
Sex			
Male	2,318 (100.0)	436 (52.3)	NA
Female	0 (0.0)	397 (47.7)	NA
Median age, years (IQR)	34 (26–43)	23 (20–28)	< 0.001
Origin			
Dutch	1,347 (58.1)	158 (19.0)	< 0.001
Asian	158 (6.8)	25 (3.0)	
Dutch-Antillean	56 (2.4)	68 (8.2)	
Eastern European	70 (3.0)	50 (6.0)	
European	251 (10.8)	36 (4.3)	
Latin American	146 (6.3)	32 (3.8)	
North African	37 (1.6)	43 (5.2)	
Sub-Sahara African	40 (1.7)	50 (6.0)	
Surinamese	114 (4.9)	342 (41.1)	
Turkish	46 (2.0)	18 (2.2)	
Other	47 (2.0)	10 (1.2)	
Unknown	6 (0.3)	1 (0.12)	
Symptoms at triage	1,249 (53.9)^c^	559 (67.1)	< 0.001
Notified by sex partner	683 (29.5)^d^	218 (26.2)	0.09
Sex worker (MSM or women)	68 (2.9)^e^	73 (18.4)^f^	< 0.001
Median number of sex partners in the previous six months (IQR)	8 (4–15)	3 (2–5)	< 0.001
HIV status			
Negative	1,377 (59.4)	805 (96.6)	< 0.001
Positive	900 (38.8)	7 (0.84)	
Unknown	41 (1.8)	21 (2.5)	
Previous or active syphilis			
No	1,566 (67.6)	819 (98.3)	< 0.001
Yes	752 (32.4)	14 (1.7)	
*Chlamydia trachomatis* co-infection			
No	1,816 (78.3)	460 (55.2)	< 0.001
Yes	502 (21.7)	373 (44.8)	
Included anatomical site, azithromycin analysis^g^			
Urethra	752 (32.4)	451 (54.1)	< 0.001
Rectum	1,301 (56.1)	64 (7.7)	
Cervix^h^	NA	263 (31.6)	
Pharynx	265 (11.4)	55 (6.6)	
Included anatomical site, ceftriaxone analysis^g^			
Urethra	740 (31.9)	451 (54.1)	< 0.001
Rectum	1,305 (56.3)	80 (9.6)	
Cervix^h^	NA	252 (30.3)	
Pharynx	273 (11.8)	50 (6.0)	
Number of culture positive sites			
1	2,098 (90.5)	704 (84.5)	< 0.001
2	218 (9.4)	109 (13.1)	
3	2 (0.1)	18 (2.2)	
4	NA	2 (0.2)	

HIV: human immunodeficiency virus; IQR: interquartile range; MIC: minimum inhibitory concentration; MSM: men who have sex with men; NA: not applicable; STI: sexually transmitted infection.

^a^ n (%) unless otherwise indicated.

^b^ Inclusion up to and including September 2015.

^c^ For two patients the information in question was not available.

^d^ For four patients the information in question was not available.

^e^ For 25 patients the information in question was not available.

^f^ The number (n=73) and percentage (18.4%) are only presented for women, as, within the study, only one heterosexual male reported sex work.

^g^ In case of multiple infected sites per patient, the isolate with the highest MIC for was selected. Therefore the included anatomical sites differ per antimicrobial drug.

^h^ Including cervical and vaginal samples.

### Antimicrobial resistance according to European committee on antimicrobial susceptibility testing

**Figure 1:**
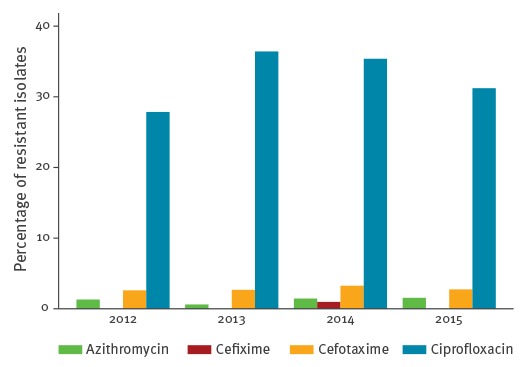
Percentage of resistant *Neisseria gonorrhoeae* isolates^a^ per year, at the STI Outpatient Clinic Amsterdam, the Netherlands, January 2012–September 2015 (n = 3,151 isolates)

EUCAST: European committee on antimicrobial susceptibility testing; MIC: minimum inhibitory concentration; STI: sexually transmitted infection.

Figure 1 shows the percentage of the 3,151 isolates that were resistant to azithromycin, cefixime, cefotaxime, and ciprofloxacin, according to EUCAST breakpoints [19]. No resistance to ceftriaxone was observed. Resistance to cefixime was rare (8 isolates in 2014, 0.3%). Overall resistance was highest for ciprofloxacin (n = 1,030, 32.7%), followed by cefotaxime (n = 89, 2.8%), and azithromycin (n = 38, 1.2%).

Azithromycin resistance: MIC > 0.5 mg/L, cefixime resistance: MIC > 0.125 mg/L, cefotaxime resistance: MIC > 0.125 mg/L, ciprofloxacin resistance: MIC > 0.06 mg/L.

^a^ According to EUCAST breakpoints.

### Azithromycin susceptibility

**Figure 2:**
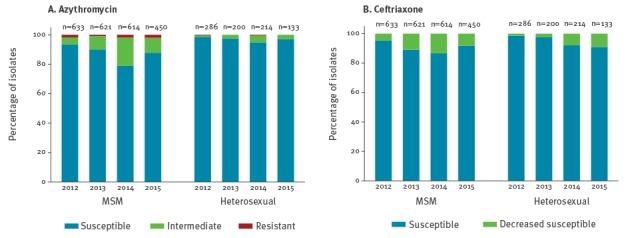
Susceptibility to azithromycin and ceftriaxone of *Neisseria gonorrhoeae* isolates, according to year and sexual orientation, STI Outpatient Clinic Amsterdam, the Netherlands, January 2012–September 2015 (n = 3,151 isolates)

The mean azithromycin MIC was 0.12 mg/L, with a range of < 0.016 to > 256 mg/L (Table 2). When categorising according to EUCAST breakpoints, overall 2,838 of the 3,151 isolates (90.1%) were susceptible, 275 (8.7%) were intermediate, and 38 (1.2%) were resistant [19]. Over time the mean MIC increased from 0.09 mg/L in 2012 to 0.13 mg/L in 2015, and the percentage of resistant strains increased slightly from 1.3% (12/919) in 2012 to 1.5% (9/583) in 2015. However, the percentage of intermediate MICs increased from 3.7% (34/919) to 8.6% (50/583), especially among MSM (Figure 2).

**Table 2:** Susceptibility to azithromycin and ceftriaxone by year of infection, of *Neisseria gonorrhoeae* isolates from the STI Outpatient Clinic Amsterdam, the Netherlands, January 2012–September 2015 (n = 3,151 isolates)

Antibiotic and characteristics of the isolates	Total 3,151	Year and number of isolates			
		2012 919	2013 821	2014 828	2015 583
**Azithromycin**					
Mean^a^ MIC in mg/L (range)	0.12 (< 0.016 to > 256)	0.09 (< 0.016 to > 256)	0.12 (< 0.016–4)	0.15 (< 0.016 to > 256)	0.13 (< 0.016–64)
Susceptible: MIC ≤ 0.25 mg/L; n(%)	2,838 (90.1)	873 (95.0)	754 (91.8)	687 (83.0)	524 (89.9)
Intermediate: MIC > 0.25 to ≤ 0.5 mg/L; n (%)	275 (8.7)	34 (3.7)	62 (7.6)	129 (15.6)	50 (8.6)
Resistant: MIC > 0.5 mg/L; n (%)	38 (1.2)	12 (1.3)	5 (0.6)	12 (1.5)	9 (1.5)
**Ceftriaxone**					
Mean MIC^a^ in mg/L (range)	0.005 (< 0.002–0.125)	0.004 (< 0.002–0.094)	0.006 (< 0.002–0.125)	0.007 (< 0.002–0.125)	0.005 (< 0.002–0.125)
Susceptible: MIC ≤ 0.032 mg/L; n (%)	2,898 (92.0)	886 (96.4)	748 (91.1)	730 (88.2)	534 (91.6)
Decreased susceptible: MIC > 0.032 mg/L; n(%)	253 (8.0)	33 (3.6)	73 (8.9)	98 (11.8)	49 (8.4)

MIC: minimum inhibitory concentration, STI: sexually transmitted infection.

^a^ Mean was calculated as geometric mean.

MIC: minimum inhibitory concentration, MSM: men who have sex with men, STI: sexually transmitted infection.

Azithromycin susceptible: MIC ≤ 0.25 mg/L, intermediate: MIC 0.38–0.5 mg/L, resistant: MIC > 0.5 mg/L. Ceftriaxone susceptible: MIC ≤ 0.032 mg/L, decreased susceptible: MIC > 0.032 mg/L.

### Determinants of decreased azithromycin susceptibility (MIC > 0.25 mg/L)

#### Men who have sex with men

Decreased susceptibility to azithromycin was 12.5% (289/2,318). Univariable logistic regression analysis (Table 3) showed an association (p < 0.1) between decreased susceptibility and year of infection, anatomical site, number of infected anatomical sites, and HIV-status. In the multivariable analysis decreased susceptibility was significantly associated with infections diagnosed in 2014 (odds ratio (OR): 3.83; 95%-confidence interval (CI): 2.64–5.55, compared with 2012), and two infected sites (OR: 1.56; 95% CI: 1.05–2.30), and was less frequent in HIV-positive patients (OR: 0.72; 95% CI: 0.54–0.96).

**Table 3:** Determinants, according to logistic regression analysis, of decreased susceptibility for azithromycin (MIC > 0.25 mg/L) and ceftriaxone (MIC > 0.032 mg/L) in *Neisseria gonorrhoeae* isolates from men who have sex with men at the STI Outpatient Clinic Amsterdam, the Netherlands, 2012–2015 (n = 2,318 isolates)

Characteristics	Azithromycin					Ceftriaxone				
	N (%)	OR (95% CI)	P	aOR (95% CI)	P	N (%)	OR (95% CI)	P	aOR (95% CI)	P
Year of diagnosis										
2012	42 (6.6)	1.00	< 0.001	1.00	< 0.001	29 (4.6)	1.00	< 0.001	1.00	< 0.001
2013	62 (10.0)	1.56 (1.04–2.35)		1.57 (1.04–2.37)		68 (11.0)	2.56 (1.63–4.02)		2.56 (1.63–4.02)	
2014	130 (21.2)	3.78 (2.62–5.46)		3.83 (2.64–5.55)		81 (13.2)	3.17 (2.04–4.91)		3.00 (1.92–4.66)	
2015^a^	55 (12.2)	1.96 (1.29–2.99)		1.93 (1.26–2.95)		37 (8.2)	1.87 (1.13–3.08)		1.71 (1.03–2.83)	
Age in years										
≤ 24	55 (12.9)	1.00	0.82	1.00	0.74	49 (11.5)	1.00	0.34	1.00	0.35
25–34	106 (13.1)	1.02 (0.72–1.44)		1.18 (0.82–1.68)		69 (8.5)	0.72 (0.49–1.06)		0.73 (0.49–1.08)	
35–44	72 (11.6)	0.88 (0.61–1.28)		1.15 (0.78–1.71)		53 (8.5)	0.72 (0.47–1.08)		0.79 (0.52–1.19)	
≥ 45	56 (12.2)	0.93 (0.63–1.39)		1.26 (0.83–1.92)		44 (9.5)	0.81 (0.53–1.25)		0.95 (0.61–1.47)	
Origin										
Dutch	155 (11.5)	1.00	0.25	Excluded^b^	NA	121 (9.0)	1.00	0.74	Excluded^b^	NA
Non-Dutch	133 (13.8)	1.23 (0.96–1.58)		Excluded^b^		93 (9.6)	1.08 (0.81–1.44)		Excluded^b^	
Unknown	1 (16.7)	1.54 (0.18–13.3)		Excluded^b^		1 (16.7)	2.03 (0.23–17.45)		Excluded^b^	
Anatomical site										
Urethra	95 (12.6)	1.00	0.02	Excluded^b^	NA	53 (7.2)	1.00	< 0.001	1.00	< 0.001
Rectum	147 (11.3)	0.88 (0.67–1.16)		Excluded^b^		117 (9.0)	1.28 (0.91–1.79)		1.29 (0.92–1.82)	
Pharynx	47 (17.7)	1.49 (1.02–2.18)		Excluded^b^		45 (16.5)	2.56 (1.67–3.91)		2.52 (1.64–3.89)	
Number of sex partners^c,d^										
0–2	35 (12.3)	1.00	0.82	Excluded^b^	NA	25 (8.8)	1.00	0.79	Excluded^b^	NA
3–6	98 (12.5)	1.02 (0.67–1.54)		Excluded^b^		71 (9.1)	1.03 (0.64–1.67)		Excluded^b^	
7–15	84 (11.7)	0.95 (0.62–1.44)		Excluded^b^		73 (10.2)	1.18 (0.73–1.89)		Excluded^b^	
≥ 16	72 (13.5)	1.11 (0.72–1.72)		Excluded^b^		46 (8.7)	0.98 (0.59–1.63)		Excluded^b^	
HIV status										
Negative	191 (13.9)	1.00	0.02	1.00	0.04	144 (10.5)	1.00	0.05	Excluded^b^	NA
Positive	91 (10.1)	0.70 (0.54–0.91)		0.72 (0.54–0.96)		67 (7.4)	0.69 (0.51–0.93)		Excluded^b^	
Missing	7 (17.1)	1.28 (0.56–2.93)		1.43 (0.62–3.33)		4 (9.8)	0.93 (0.33–2.63)		Excluded^b^	
Previous or active syphilis										
No	196 (12.5)	1.00	0.92	Excluded^b^	NA	158 (10.1)	1.00	0.05	Excluded^b^	NA
Yes	93 (12.4)	0.99 (0.76–1.28)		Excluded^b^		57 (7.6)	0.73 (0.53–1.00)		Excluded^b^	
*Chlamydia trachomatis*										
No	225 (12.4)	1.00	0.83	Excluded^b^	NA	172 (9.5)	1.00	0.53	Excluded^b^	NA
Yes	64 (12.8)	1.03 (0.77–1.39)		Excluded^b^		43 (8.6)	0.90 (0.63–1.27)		Excluded^b^	
Number of anatomical sites with gonorrhoea										
1	253 (12.1)	1.00	0.07	1.00	0.03	193 (9.2)	1.00	0.67	Excluded^b^	NA
2	36 (16.5)	1.44 (0.99–2.11)		1.56 (1.05–2.30)		22 (10.1)	1.11 (0.70–1.76)		Excluded^b^	
3	0 (0.0)	NA		NA		0 (0.0)	NA		Excluded^b^	

aOR: adjusted odds ratio; CI: confidence interval; HIV: human immunodeficiency virus; MIC: minimum inhibitory concentration; n: number of isolates with decreased susceptibility; NA: not applicable; OR: odds ratio; STI: sexually transmitted infection.

^a^ Inclusion up to and including September 2015.

^b^ Variable that was excluded by backward selection.

^c^ In previous six months.

^d^ Four patients with information on number of sexual partners missing.

#### Heterosexuals

The percentage of isolates with decreased susceptibility to azithromycin in heterosexuals was 2.9% (24/833), which was significantly lower compared with MSM (p < 0.001). Univariable logistic regression analysis (Table 4) showed an association (p < 0.1) with sex, age, origin, and number of sex partners. Higher ORs were observed for calendar years after 2012 (p = 0.11). In the multivariable regression only ≥ 10 sex partners in the previous six months was significantly associated with decreased susceptibility (OR: 5.65; 95% CI: 1.49–21.39, compared with 0–1 sex partners).

**Table 4:** Determinants, according to logistic regression analysis, of decreased susceptibility to azithromycin (MIC > 0.25 mg/L) and ceftriaxone (MIC > 0.032 mg\L) in *Neisseria gonorrhoeae* isolates from heterosexual males and females at the STI Outpatient Clinic Amsterdam, the Netherlands, 2012–2015 (n = 833 isolates)

Characteristics	Azithromycin					Ceftriaxone				
	n (%)	OR (95% CI)	p	aOR (95% CI)	p	n (%)	OR (95% CI)	p	aOR (95% CI)	p
Year of diagnosis										
2012	4 (1.4)	1.00	0.11	1.00	0.35	4 (1.4)	1.00	< 0.001	1.00	< 0.001
2013	5 (2.5)	1.81 (0.48–6.82)		1.44 (0.37–5.61)		5 (2.5)	1.81 (0.48–6.82)		1.12 (0.28–4.44)	
2014	11 (5.1)	3.82 (1.20–12.17)		2.74 (0.83–9.11)		17 (7.9)	6.08 (2.02–18.36)		5.44 (1.71–17.23)	
2015^a^	4 (3.0)	2.19 (0.54–8.88)		1.65 (0.38–7.15)		12 (9.0)	6.99 (2.21–22.11)		5.54 (1.65–18.65)	
Sex										
Male	8 (1.8)	1.00	0.06	1.00	0.16	10 (2.3)	1.00	< 0.001	1.00	0.007
Female	16 (4.0)	2.25 (0.95–5.31)		1.95 (0.76–5.01)		28 (7.1)	3.23 (1.55–6.74)		3.14 (1.32–7.45)	
Age in years										
≤ 19	3 (1.8)	1.00	0.02	1.00	0.08	2 (1.2)	0.14 (0.03–0.65)	0.02	0.23 (0.05–1.17)	0.26
20–24	8 (2.5)	1.40 (0.37–5.34)		1.23 (0.31–4.84)		14 (4.4)	0.54 (0.25–1.19)		0.69 (0.28–1.70)	
25–29	2 (1.1)	0.61 (0.10–3.68)		0.51 (0.08–3.28)		9 (5.0)	0.62 (0.26–1.49)		0.77 (0.29–2.08)	
≥ 30	11 (6.6)	3.83 (1.05–13.99)		2.86 (0.71–11.60)		13 (7.8)	1.00		1.00	
Origin										
Dutch	7 (4.4)	1.00	0.09	Excluded^b^	NA	8 (5.1)	1.00	0.002	1.00	0.05
Surinamese	5 (1.5)	0.32 (0.10–1.02)		Excluded^b^		6 (1.8)	0.33 (0.11–0.98)		0.96 (0.29–3.14)	
Other	12 (3.6)	0.81 (0.31–2.10)		Excluded^b^		24 (7.2)	1.46 (0.64–3.33)		2.46 (0.98–6.21)	
Unknown	0 (0.0)	NA		Excluded^b^		0 (0.0)	NA		Excluded^b^	
Anatomical site										
Urethra	9 (2.0)	1.00	0.19	Excluded^b^	NA	11 (2.4)	1.00	< 0.001	Excluded^b^	NA
Rectum	3 (4.7)	2.42 (0.64–9.17)		Excluded^b^		10 (12.5)	5.71 (2.34–13.95)		Excluded^b^	
Cervix	8 (3.0)	1.54 (0.59–4.04)		Excluded^b^		8 (3.2)	1.31 (0.52–3.30)		Excluded^b^	
Pharynx	4 (7.3)	3.85 (1.15–12.96)		Excluded^b^		9 (18.0)	8.78 (3.44–22.42)		Excluded^b^	
Number of sex partners^c^										
0–1	3 (1.6)	1.00	< 0.001	1.00	0.01	4 (2.2)	1.00	< 0.001	1.00	0.001
2	4 (2.0)	1.21 (0.27–5.47)		1.44 (0.31–6.66)		3 (1.5)	0.67 (0.15–3.04)		0.85 (0.18–3.95)	
3–9	5 (1.6)	0.95 (0.23–4.04)		1.12 (0.26–4.84)		11 (3.4)	1.60 (0.50–5.08)		1.98 (0.59–6.66)	
≥ 10	12 (10.0)	6.74 (1.86–24.42)		5.65 (1.49–21.39)		20 (16.7)	9.05 (3.01–27.21)		6.16 (1.92–19.79)	
HIV status										
Negative	23 (2.9)	1.00	0.19	Excluded^b^	NA	38 (4.7)	NA	NA	Excluded^b^	NA
Positive	1 (14.3)	5.67 (0.66–49.00)		Excluded^b^		0 (0.0)	NA		Excluded^b^	
Missing	0 (0.0)	NA		Excluded^b^		0 (0.0)	NA		Excluded^b^	
Previous or active syphilis										
No	23 (2.8)	1.00	0.41	Excluded^b^	NA	37 (4.5)	1.00	0.66	Excluded^b^	NA
Yes	1 (7.1)	2.66 (0.33–21.22)		Excluded^b^		1 (7.1)	1.63 (0.21–12.76)		Excluded^b^	
*Chlamydia trachomatis*										
No	17 (3.7)	1.00	0.11	Excluded^b^	NA	25 (5.4)	1.00	0.18	Excluded^b^	NA
Yes	7 (1.9)	0.50 (0.20–1.21)		Excluded^b^		13 (3.5)	0.63 (0.32–1.25)		Excluded^b^	
Number of anatomical sites with gonorrhoea										
1	18 (2.6)	1.00	0.45	Excluded^b^	NA	27 (3.8)	1.00	0.07	Excluded^b^	NA
2	5 (4.6)	1.83 (0.67–5.04)		Excluded^b^		9 (8.3)	2.26 (1.03–4.94)		Excluded^b^	
3	1 (5.6)	2.24 (0.28–17.78)		Excluded^b^		1 (5.6)	1.47 (0.19–11.49)		Excluded^b^	
4	0 (0.0)	NA		Excluded^b^		1 (50.0)	25.07 (1.53–411.66)		Excluded^b^	

HIV: human immunodeficiency virus; MIC: minimum inhibitory concentration; n: number of isolates with decreased susceptibility; NA: not applicable; OR: odds ratio; aOR: adjusted odds ratio; 95%-CI: 95% confidence interval; STI: sexually transmitted infection.

^a^ Inclusion up to and including September 2015.

^b^ Variable that was excluded by backward selection.

^c^ In the previous six months.

### Ceftriaxone susceptibility

The mean MIC was 0.005 mg/L, the range was < 0.002–0.125 mg/L (Table 2). We categorised 2,898 of the 3,151 isolates (92.0%) as susceptible (MIC ≤ 0.032 mg/L), and 253 isolates (8.0%) as decreased susceptible (MIC > 0.032 mg/L). The mean MIC increased slightly from 0.004 mg/L in 2012, to 0.005 mg/L in 2015. The percentage of decreased susceptible isolates increased from 3.6% (33/919) in 2012 to 8.4% (49/583) in 2015. This increase was noted among both MSM and heterosexuals (Figure 2).

### Determinants of ceftriaxone decreased susceptibility (MIC > 0.032 mg/L)

#### Men who have sex with men

The percentage of isolates with decreased susceptibility to ceftriaxone in MSM was 9.3% (215/2,318). Univariable logistic regression analysis (Table 3) showed an association (p < 0.1) between decreased susceptibility and calendar year, anatomical site of infection, HIV-status, and previous or active syphilis. In the multivariable analysis decreased susceptibility was significantly associated with infections diagnosed in 2014 (OR: 3.00, 95% CI: 1.92–4.66, compared with 2012), and pharyngeal infection (OR: 2.52, 95% CI: 1.64–3.89, compared with urethral infection).

#### Heterosexuals

The percentage of isolates with decreased susceptibility to ceftriaxone in heterosexuals was 4.5% (38/833), which was significantly lower compared with MSM (p < 0.001). Univariable logistic regression analysis (Table 4) showed an association (p < 0.1) with year of infection, sex, age, origin, anatomical site of infection, number of sex partners, and number of infected anatomical sites. In the multivariable analysis infections diagnosed in 2014 (OR: 5.44; 95% CI: 1.71–17.23, compared with 2012), female sex (OR: 3.14; 95% CI: 1.32–7.45), and ≥ 10 sex partners (OR: 6.16; 95% CI: 1.92–19.79, compared with 0–1 sex partners) were significantly associated with decreased susceptibility.

### Decreased susceptibility to azithromycin or ceftriaxone, and resistance to other drugs

Among the 313 isolates with decreased susceptibility for azithromycin, 110 isolates (35.1%) were resistant to ciprofloxacin, 20 (6.4%) to cefotaxime and two (0.6%) to cefixime. In addition, 18 isolates (5.8%) were resistant to at least two antibiotics (apart from azithromycin). Among the 253 isolates with decreased susceptibility to ceftriaxone, 242 (95.7%) were resistant to ciprofloxacin, 80 (31.6%) to cefotaxime, six (2.4%) to azithromycin, and six (2.4%) to cefixime. Also 72 isolates (28.5%) were resistant to at least two, and eight (3.2%) to at least three antibiotics (apart from ceftriaxone).

## Discussion

This study shows trends in antimicrobial resistance, and determinants of decreased susceptibility for azithromycin and ceftriaxone in *N. gonorrhoeae* at the STI Clinic Amsterdam, the Netherlands, from January 2012 through September 2015. Resistance to azithromycin remained stable around 1.2%, although the percentage of isolates with intermediate MICs increased from 3.7% in 2012 to 15.6% in 2014, and then decreased to 8.6% in the first nine months of 2015. Resistance to ceftriaxone has not yet been documented in our population. Decreased susceptibility to ceftriaxone (defined as MIC > 0.032 mg/L) increased from 3.6% in 2012 to 11.8% in 2014, and then decreased to 8.4% in the first nine months of 2015. Future surveillance will demonstrate if these small decreases in reduced susceptibility continue, and if so may provide reasons for this. Like we published previously in 2009, decreased susceptibility or resistance to more than one drug remains common [3]. Among isolates with decreased susceptibility to azithromycin or ceftriaxone, 35.1% and 95.7% respectively were also resistant to ciprofloxacin.

Compared with data of various other European countries as reported by the European Centre for Disease Prevention and Control (ECDC), overall resistance in Amsterdam is lower [20]. Although overall resistance was highest for ciprofloxacin (32.7%), it is lower than the overall European prevalence of ciprofloxacin resistance (53%) reported from 2012 to 2013 [20,21]. An explanation could be the large inter-country variability, and the large number of MSM in our population, as in Europe ciprofloxacin resistance was most common among heterosexual males [20]. Cefixime resistance across Europe is 5% [20-22]. Our results show lower cefixime resistance in Amsterdam (0.3%; only noted in 2014), which is comparable to that in the United States from 2006 to 2014 [23]. Cefixime has never been used as first-line treatment of gonorrhoea in the Netherlands, which could explain the lower prevalence of cefixime resistance in our population. Due to unavailability of ceftriaxone in required dosages, cefotaxime was the first-line treatment in the Netherlands for several years up to 2006, which may have caused the relatively high overall resistance for cefotaxime (2.8%) in Amsterdam [3,24]. Since cefotaxime was abandoned as first-line treatment, resistance has decreased again from 12% at the end of 2008, to 2.7% in 2015 [3,25]. Ceftriaxone resistance has been reported in the WHO Western Pacific Region, Asia, the United States and also in several European countries [20,22,23,26,27]. Despite the concurrent increase of ceftriaxone resistance, no resistant isolates have been documented in the Netherlands yet [20,22,23,26,27]. European azithromycin resistance is reported at 5% in 2013 [20,21,28]. In our population azithromycin resistance has not been above 1.5% since 2012, which is lower than the overall European prevalence. Although both the mean MIC and the percentage of resistance have increased slightly during our study period, the high increase reported elsewhere in Europe, was not seen in our population [9,20]. The outbreak of azithromycin high-resistant isolates in England in 2015 occurred despite the use of dual therapy, as recommended by European and United States Centers for Disease Control and Prevention (CDC) guidelines [4,5]. Dutch guidelines do not recommend dual therapy, but advise a single intramuscular dose of 500 mg ceftriaxone [17]. Azithromycin is only added if a *C. trachomatis* coinfection is suspected or diagnosed. The strict adherence to the Dutch guidelines at our clinic will have resulted in lower exposure of our population to azithromycin. In addition, over the counter antibiotics are not available in the Netherlands, and self administration of azithromycin will have been very limited. As exposure to antibiotics is the most important risk factor for antimicrobial resistance, the lower exposure to azithromycin in our population could account for the absence of increased azithromycin resistance in Amsterdam [29,30]. However, the larger increase in isolates with an intermediate MIC during our study period suggests that an increase in resistant strains is possible in the future.

Strains with decreased susceptibility, for either azithromycin or ceftriaxone, were significantly more often isolated from MSM compared with heterosexuals (both p < 0.001). This suggest that sexual orientation (or risk behaviour) is associated with decreased susceptibility to both azithromycin and ceftriaxone. However, because of correlation with other variables, we had to stratify for sexual orientation, and could not correct this possible association for confounders. Among MSM we noted a significant association between more recent year of infection (more recent than 2012) and decreased susceptibility to both azithromycin and ceftriaxone. These results confirm the reported decrease in azithromycin and ceftriaxone susceptibility in Europe [20,26,31]. For heterosexuals being diagnosed in 2014 or 2015 compared to 2012 was only significantly associated with decreased susceptibility to ceftriaxone. Unlike in other countries, this association was not significant for azithromycin, possibly due to a lower number of samples with decreased susceptibility in this group (n = 24) [20]. In addition to time, decreased ceftriaxone susceptibility among MSM was associated with pharyngeal infections. We did not find an association with anatomical site for azithromycin, in either MSM or heterosexuals. Although studies combining antimicrobial resistance and epidemiology are few, previous studies in the UK and France also report higher ceftriaxone MICs in pharyngeal infections [26,31,32]. It is of concern that many cases of pharyngeal gonorrhoea are culture negative, resulting in no diagnosis or diagnosis by NAAT only (which is the recommended routine diagnostic test) [33]. Pharyngeal infections due to strains with decreased susceptibility or even resistance could therefore be missed by routine diagnosis. This is especially worrisome because it is assumed that ceftriaxone resistance in *N. gonorrhoeae* originates from commensal *Neisseria* species in the pharynx [14,34]. Unlike Trecker et al. and Town et al. we found not male, but female sex to be significantly associated with decreased ceftriaxone susceptibility [26,35]. This association might have been caused by the substantial number of sex workers (18%) among women in our study. However, when adjusting for the number of sex partners (a very good proxy for sex work), female sex remained significantly associated. Also, in a sensitivity analysis adjusting for sex work, female sex still remained significantly associated with decreased ceftriaxone susceptibility (data not shown). In addition, like Town et al. our study shows no significant association with age, in contrast to what was previously reported by Trecker et al. [26,35]. However, among heterosexuals, we did find a significant association between a high number of sex partners (≥ 10; this category consisted mainly of female sex workers) and decreased susceptibility to both azithromycin and ceftriaxone. This adds to the limited evidence that high risk-behaviour and the associated sexual networks are important factors for the spread of resistance among heterosexuals [22,35]. To improve surveillance in populations at high risk of resistant gonorrhoea more studies combining susceptibility and epidemiological data are needed.

There are some limitations to this study. We selected isolates based on new consultations, and some patients were included multiple times. If patients were reinfected by an untreated partner, the same strain could have been included more than once. Depending on the susceptibility of such a strain this could have influenced our analysis of determinants for decreased susceptibility. The change in policy to obtain cultures at the STI clinic in May 2014 may have changed the composition of patients in our study population, and thus could have influenced our results. MSM and commercial sex workers were no longer primarily tested using culture, but with NAAT. In addition, cultures were mainly obtained from patients returning to the STI clinic for treatment after a positive NAAT. Therefore, cultures from patients who did not return to the STI clinic, or did not consent to sampling for culture may have been missed after May 2014. Lastly, as we did not have information on the use of alcohol or drugs, or travel history from our population. Therefore, we were unable to take these possible determinants of decreased susceptibility into account [35,36].

In conclusion, between 2012 and 2015 antimicrobial resistance to azithromycin was less prevalent in Amsterdam compared with other European countries. However, we did note a rise in decreased susceptibility, particularly among MSM. Resistance to ceftriaxone has not been documented in the Netherlands yet, but we also noted a rise in decreased ceftriaxone susceptibility among both MSM and heterosexuals. Given the higher resistance in other countries and increasing globalisation, standardised surveillance of antimicrobial resistance in *N. gonorrhoeae* will remain indispensable. A continued and combined increase of azithromycin and ceftriaxone resistance will likely impede the effectivity of the current dual therapy. Because there is very limited development of new antibiotics, this could lead to severe public health consequences, such as hospital admittance for intravenous treatment in patients with gonorrhoea. Therefore, urgency in the development of novel treatment strategies and reassessment of older antimicrobial agents is warranted. Funding for this research is essential on both national and European levels.
